# Genome-Wide Identification and Analysis of Gene Family of Carbohydrate-Binding Modules in *Ustilago crameri*

**DOI:** 10.3390/ijms252111790

**Published:** 2024-11-02

**Authors:** Dongyu Zhai, Deze Xu, Ting Xiang, Yu Zhang, Nianchen Wu, Fuqing Nie, Desuo Yin, Aijun Wang

**Affiliations:** 1 College of Agronomy, Guangxi University, Nanning 530004, China; m19555726879@163.com (D.Z.); xt18728783187@163.com (T.X.); 2 College of Plant Protection, Henan Agricultural University, Zhengzhou 475004, China; 18436618796@163.com (Y.Z.); 15903059619@163.com (N.W.); 18037822703@163.com (F.N.); 3 Food Crop Research Institute, Hubei Academy of Agriculture Sciences, Wuhan 430062, China; dezexu@163.com

**Keywords:** *Ustilago crameri*, foxtail millet, CBM, pathogenicity

## Abstract

*Ustilago crameri* is a pathogenic basidiomycete fungus that causes foxtail millet kernel smut (FMKS), a devastating grain disease in most foxtail millet growing regions of the world. Carbohydrate-Binding Modules (CBMs) are one of the important families of carbohydrate-active enzymes (CAZymes) in fungi and play a crucial role in fungal growth and development, as well as in pathogen infection. However, there is little information about the CBM family in *U. crameri*. Here, 11 CBM members were identified based on complete sequence analysis and functional annotation of the genome of *U. crameri*. According to phylogenetic analysis, they were divided into six groups. Gene structure and sequence composition analysis showed that these 11 UcCBM genes exhibit differences in gene structure and protein motifs. Furthermore, several cis-regulatory elements involved in plant hormones were detected in the promoter regions of these UcCBM genes. Gene ontology (GO) enrichment and protein–protein interaction (PPI) analysis showed that UcCBM proteins were involved in carbohydrate metabolism, and multiple partner protein interactions with UcCBM were also detected. The expression of UcCBM genes during *U. crameri* infection is further clarified, and the results indicate that several UcCBM genes were induced by *U. crameri* infection. These results provide valuable information for elucidating the features of *U. crameri* CBMs’ family proteins and lay a crucial foundation for further research into their roles in interactions between *U. crameri* and foxtail millet.

## 1. Introduction

Plant pathogenic fungi can degrade host cell walls’ polysaccharide materials by producing a variety of carbohydrate activity enzymes (CAZymes) to facilitate infection and gain nutrition [1]. Carbohydrate-binding modules (CBMs) are widely present in the microorganism, and are important components of the CAZymes of plant pathogenic fungi [2]. CBMs are the most common non-catalytic modules associated with active cell wall hydrolysis enzymes, and they are divided into 87 families in the CAZymes database [3,4]. This protein family plays a key role in the recognition of plant cell wall components and in enhancing the enzyme activity of glycoside hydrolases [5].

More importantly, the roles of CBMs in the pathogenicity of plant pathogens have been confirmed [6,7]. For example, the CBM1 domain protein VdCBM1 from *Verticillium dahliae* suppressed VdEG1 and VdEG3 protein-induced cell death in *Nicotiana benthamiana* [8]. The CBM protein PcCBP3, which comes from the oomycete pathogen *Phytophthora capsica*, was found to be a crucial virulence factor and play a crucial role as an apoplastic effector in interactions between *P. capsica* and its host [9]. Furthermore, deletion of the swollenin gene that contains an N-terminal CBM1 and C-terminal expansin-like domain results in the significantly reduced virulence of *Trichoderma reesei* on the host plant [10]. These findings suggest that CBMs are important virulence factors in phytopathogens; the study of CBM proteins would be beneficial to the study of the molecular mechanisms of interactions between pathogens and plants.

Foxtail millet (*Setaria italica* L.) is one of the world’s oldest cultivated crops, providing the daily dietary intake for millions of people in southern Europe and Asia [11]. *Ustilago crameri* is an important pathogenic fungus affecting the production of foxtail millet in the world [11]. This disease was first reported in Uttarakhand and then found in India, Karnataka, Andhra Pradesh, Tamil Nadu Maharashtra, and China [11,12,13,14]. A major feature of this pathogen is that it affects grains by producing a dark black powdery mass of spores in grain ears; however, sometimes, a terminal portion of the spike may escape [15]. The ideal soil temperature for infection ranges from 12 to 25 °C; whether the soil is dry or has high-humidity will significantly impact the *U. crameri* infection [12]. *U. crameri* can infect foxtail millet at all growth stages and results in a high incidence of infection of 75% in severe years, leading to a significant decrease in foxtail millet yield [11]. This disease is now an increasing threat to the high production of foxtail millet in most foxtail millet production areas. Previous studies have focused on the biological characteristics of *U. crameri* [11], while little is known about the pathogenic genes of this pathogen.

As of recently, the completion of *U. crameri* genome sequencing has offered necessary tools for researching pathogenesis at the level of genomes, and has supported the feasibility of performing the large-scale cloning of pathogenicity genes [16,17]. In this study, we identified 11 CBM family genes by searching the genome of *U. crameri* SCZ-6 using a bioinformatics analysis. Their physicochemical protein properties, conserved motif, gene structure, and promoter cis-acting elements were analyzed. We also detected the expression pattern of these 11 CBM genes during *U. crameri* infection and constructed their corresponding protein–protein interaction network. The results of this study promote a deeper understanding of CBM genes in *U. crameri* and provide a theoretical basis for exploring their roles in the pathogenesis of *U. crameri*.

## 2. Results

### 2.1. Identification and Characterization of UcCBM Gene Family

We identified a total of 11 CBM members in *U. crameri*, which were named UcCBM1-UcCBM11, and these proteins belong to the superfamilies CBM48, CBM4, CBM43, CBM18, CBM21, CBM63, CBM35, CBM50, and CBM32, respectively (Table 1). The protein length, molecular weight, isoelectric points (PIs), grand average of hydropathicity (GRAVH), and subcellular localization are shown in Table 1. The protein length of these UcCBMs ranged from 171 amino acids (aas) (UcCBM11) to 1007 aas (UcCBM5), corresponding to protein molecular weights of 12.14 and 88.78 kDa, respectively. The PI of UcCBM8 was the lowest with 4.57, and the highest was UcCBM4 with 8.83, and the mean isoelectric point of these 11 CBM proteins was 6.18, which indicates acidity. These results indicate that UcCBMs have a wide range of PIs and molecular weights. The GRAVH of these UcCBM proteins was negative, except for UcCBM3, indicating that the majority of UcCBM proteins are associated with hydrophilicity. In addition, the subcellular localization of UcCBM proteins was predicted, and the results showed that the majority of UcCBM proteins were localized extracellularly, indicating that they function primarily in the extracellular space (Table 1).

### 2.2. Phylogenetic Analysis of UcCBM Gene Family

**Figure 1:**
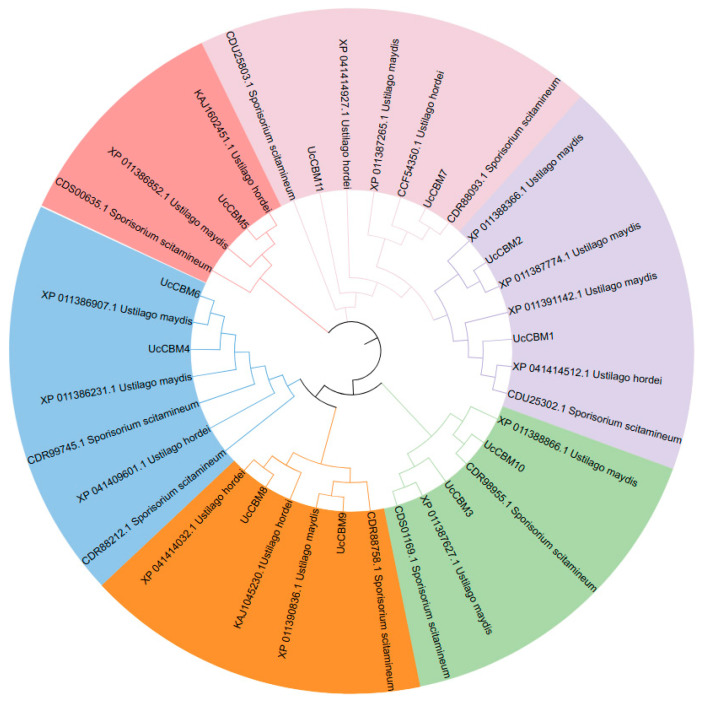
A phylogenetic tree of the UcCBM proteins and their homologous proteins in other smut fungi, including *Sporisorium scitamineum*, *U. maydis*, and *U. hordei*. The phylogenetic tree was constructed with MEGA7 software using the Neighbor-Joining (NJ) method. Different groups are represented by branches and frames of different colors.

To detect the phylogenetic relationship of CBM family proteins in *U. crameri*, a phylogenetic tree of these UcCBM proteins and their homologous proteins in other smut fungi, including *Sporisorium scitamineum*, *U. maydis*, and *U. hordei*, was constructed by MEGA7.0 software. The results indicated that these 11 UcCBM proteins could be divided into six groups, among which two CMB members, UcCBM10 and UcCBM3, were in one group. The other group contained UcCBM2 and UcCBM1, and UcCBM7 and UcCBM11 were classified in the same group. Furthermore, UcCBM4 and UcCBM6 had a close relationship, and UcCBM5 was in a separate group (Figure 1). All of the above information shows that these 11 UcCBM proteins have large differences in their evolution; UcCBM5 has especially distant phylogenetic relationships with the other 10 CBM proteins, indicating that this CBM protein may have a specific biological function (Figure 1).

### 2.3. Conserved Motif, Gene Structure Analysis of UcCBM Gene Family

**Figure 2:**
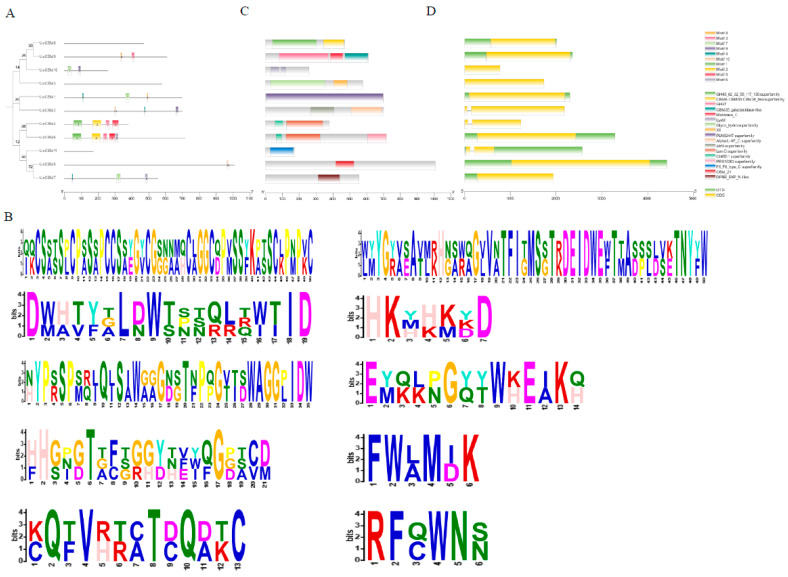
Motif distribution, conserved domain, and gene structure analysis of UcCBMs. (**A**) Phylogenetic trees and conserved motifs of 11 UcCBMs. (**B**) nserved motif sequence logo of UcCBM proteins. (**C**) Domain analysis of UcCBMs. (**D**) Exon–intron structures of UcCBMs.

To understand the characteristics of the *U. crameri* CBM family, the conserved motifs of the UcCBM proteins were analyzed using the website Multiple Em for Motif Elicitation (MEME). The results showed that 10 different motifs in the UcCBM proteins were found, named Motif 1–10 (Figure 2A,B). Among these 11 UcCBM proteins, UcCBM6 contained the highest number of motifs with five different motifs, while UcCBM11, UcCBM3, and UcCBM8 had no motif. Interestingly, we found that UcCBM6 and UcCBM4 had the same motifs, except for Motif 6, indicating that these two UcCBMs may have the same biological function during infection of a host (Figure 2A). In addition, Motif 7, Motif 4, and Motif 3 were the most common motifs, found in three UcCBM proteins (Figure 2A). Other motifs were detected in two UcCBM proteins (Figure 2A). An analysis of the functional domains found that multiple types of conserved domains related to carbohydrates were found in the 11 CBM family proteins (Figure 2B). However, only UcCBM5, UcCBM9, and UcCBM8 had the conserved domain of typical CBM or GH family proteins (Figure 2B). In addition, the structural features of the 11 UcCBM genes were further detected. As shown in Figure 2C, among 11 UcCBM genes, UcCBM2, UcCBM4, and UcCBM11 had the highest number of exons and introns; they contained two exons and one intron, respectively. However, other UcCBM genes only contained one exon and no introns. The conserved motifs of UcCBMs in the same branch were various, suggesting that these UcCBMs may play different functions in *U. cramer* virulence (Figure 2A).

### 2.4. Analysis of Cis-Acting Promoter Elements of UcCBM Gene Family

**Figure 3:**
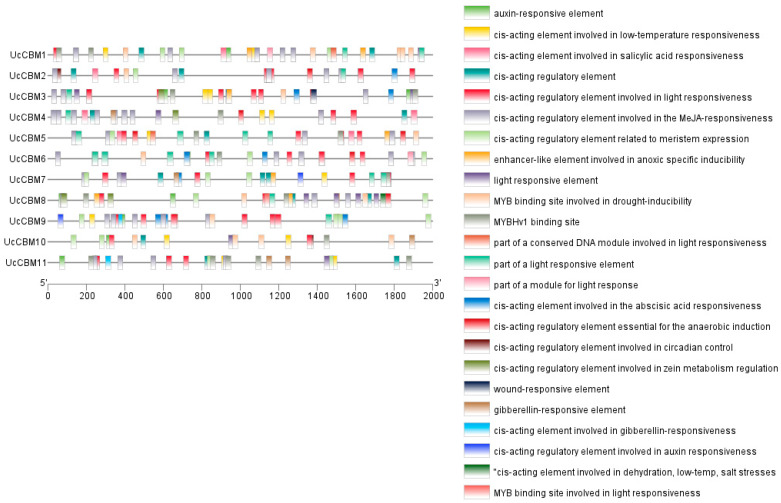
Cis-acting elements of UcCBM family members in *U. crameri*. The 2000 bp promoter sequences of *U. crameri* UcCBM genes contain a variety of cis-acting elements, including hormone responsive elements, drought, low-temperature, and other response elements, as well as an MYBHv1 binding site.

To explore the UcCBM-involved signal regulation pathways, the cis-elements in the 2000 bp sequence upstream of the 11 UcCBM gene families were analyzed. According to the results, multiple cis-elements were identified in the UcCBMs (Figure 3 and Table S1). Among them, the hormone response elements included an auxin-responsive element, salicylic acid-responsive element, MeJA-responsive elements, abscisic acid-responsive element, and gibberellin-responsive element. The latter was the most frequent in the UcCBM genes, and all 11 genes contained hormone response elements, which is the most in the UcCBM gene family, suggesting that UcCBMs may be involved in regulating the hormones of *U. crameri* (Figure 3 and Table S1). There were 7 and 8 UcCBM genes containing low-temperature and drought response elements, respectively, indicating that the UcCBMs may also play a crucial role in the mitigation of stress (Figure 3 and Table S1). Furthermore, we found that an MYBHv1 binding site was contained in 9 UcCBM genes, suggesting that the transcriptional expression of these 9 UcCBMs can be regulated by the MYB transcription factor (Figure 3 and Table S1). Therefore, these results confirm that UcCBMs may be involved in multiple biological processes, and provide important information for studying the biological function of the UcCBM gene family in *U. crameri* growth and development.

### 2.5. Protein Structure Analysis of UcCBM Gene Family Members

**Figure 4:**
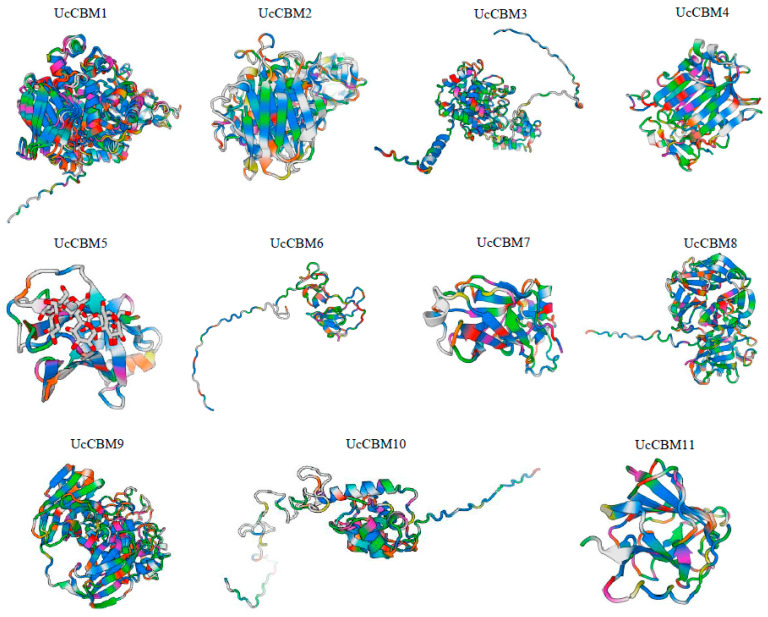
Three-dimensional (3D) modeling of the UcCBM proteins that were predicted, displayed at a confidence level > 0.7.

To better understand the biological function of CBM genes in *U. crameri*, the secondary structure of the 11 UcCBMs in *U. crameri* was detected, and the main component of their secondary structure was random coil. Specifically, alpha helices ranged from 5.42% to 32.41%, beta turns from 0.6% to 5.84%, random coil from 50.57% to 87.39%, and extended stand from 6.06% to 29.15% (Table S2). Furthermore, the three-dimensional (3D) protein structures of 11 UcCBM genes were further predicted. As shown in Figure 4, the 3D structure of these 11 UcCBM proteins exhibited substantial differences, highlighting the structural diversity prevalent within the UcCBM family.

### 2.6. Gene Ontology Enrichment Analysis of UcCBM Genes

**Figure 5:**
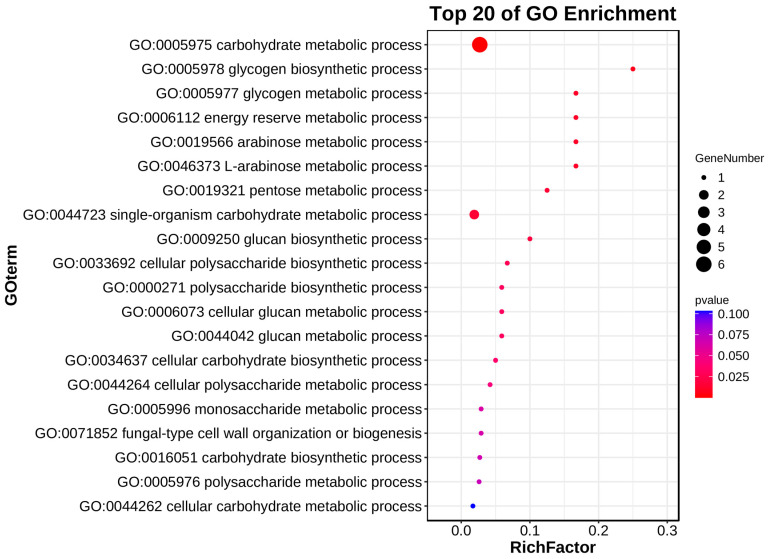
Gene Ontology enrichment analysis of *UcCBM* genes.

Gene Ontology (GO) enrichment analysis can help us understand the functions of genes in biological processes [18]. To further investigate the functions and metabolic pathways of the identified CBM proteins in *U. crameri*, GO enrichment analysis of the 11 UcCBMs was performed. The results indicated that 11 genes were enriched in 43 GO terms (Table S3). We analyzed the top 20 terms, which yielded the majority of UcCBM proteins, including UcCBM1, UcCBM2, UcCBM3, UcCBM4, UcCBM8, and UcCBM9, as having been enriched in the carbohydrate metabolic process (GO:0005975) (Figure 5). UcCBM1 was also enriched in the glycogen biosynthetic (GO:0005978), glycogen metabolic process (GO:0005977), and energy reserve metabolic process (GO:0006112) (Figure 5). UcCBM2 was enriched in the arabinose metabolic process (GO:0019566), L-arabinose metabolic process (GO:0046373), and pentose metabolic process (GO:0019321) (Figure 5). These findings show that the UcCBM genes play an important role in the carbohydrate metabolic process, especially in glycogen metabolic, arabinose metabolic, L-arabinose metabolic, and pentose metabolic processes, which are a component of plant cell walls [19].

### 2.7. Response of UcCBM Family Genes During U. crameri Infection in Foxtail Millet

**Figure 6:**
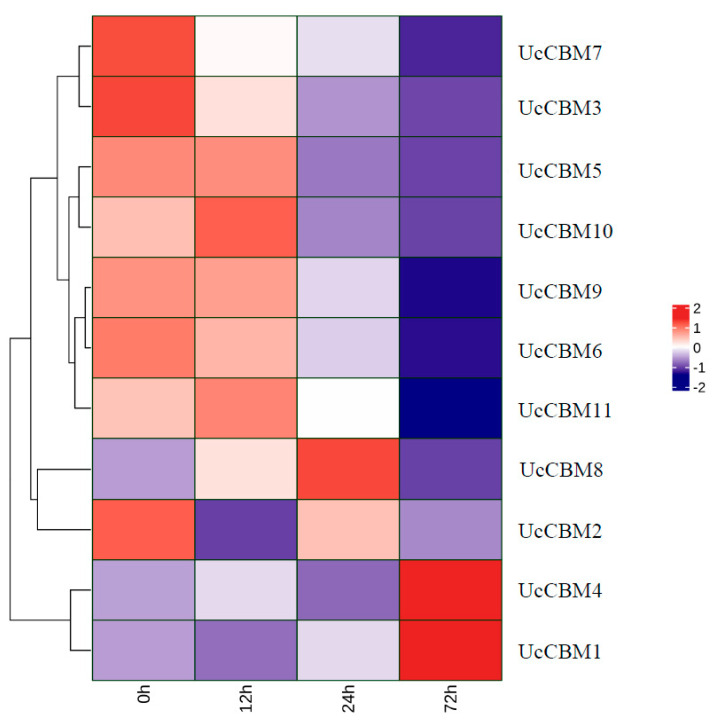
Expression of UcCBM gene in *U. crameri* at different inoculation time points.

To identify the potential candidate UcCBM genes involved in the pathogenesis of *U. crameri*, the expression of 11 UcCBM genes in *U. crameri* after inoculation was detected based on transcriptome sequencing data reported earlier [16]. The expression pattern of the 11 UcCBM family genes based on fragments per kilobase of exon model per million mapped fragments (FPKMs) values at four inoculation time points (0, 12, 24, and 72 h) is showed in Figure 6. The result indicates that the expression of six genes, UcCBM7, UcCBM3, UcCBM5, UcCBM9, UcCBM6, and UcCBM2, were downregulated after *U. crameri* inoculation, while the expression levels of UcCBM8, UcCBM4, and UcCBM1 were significantly upregulated at 48 and 72 h post-inoculation, respectively. These results confirm that UcCBM genes are involved in the pathogenicity of *U. crameri*, and play a crucial role in interactions between *U. crameri* and foxtail millet.

### 2.8. Protein−Protein Interaction Network

**Figure 7:**
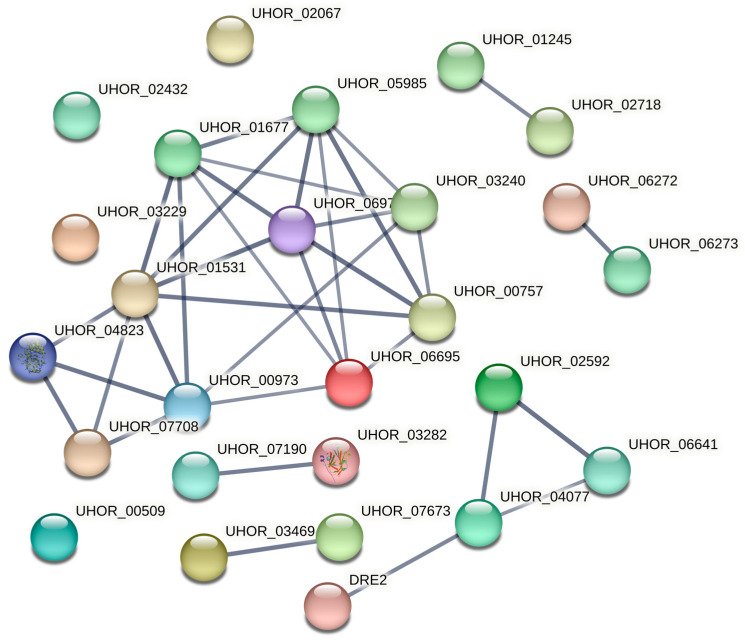
Protein–protein interaction network of UcCBM family proteins, constructed based on smut fungi *U. hordei* genome. UHOR_06976: UcCBM1; UHOR_02432: UcCBM3; UHOR_02718: UcCBM2; UHOR_00509: UcCBM4 or UcCBM6; UHOR_00973: UcCBM5; UHOR_02067: UcCBM7; UHOR_04077: UcCBM8; UHOR_06273: UcCBM9; UHOR_03469: UcCBM10; UHOR_03282: UcCBM11.

To further clarify the functional characteristics of UcCBM family proteins, the protein–protein interaction (PPI) network was analyzed using STRING v12.0. The results showed that there are 31 nodes and 53 edges, with an average node degree of 3.42 and an average local clustering coefficient of 0.645 (*p*−value< 1.0 × 10^−16^). We set the confidence level greater than 0.7 to predict the functional partner proteins of the UcCBMs, and found that multiple partner proteins interact with the UcCBMs. For instance, UcCBM1 (UHOR_06976) interacted with glycogen [starch] synthase (UHOR_01531), PGM2-phosphoglucomutase (UHOR_00757), glycogenin-2 beta (UHOR_01677), 4-alpha-glucanotransferase (UHOR_05985), and SPT14-N-acetylglucosaminyltransferase (UHOR_03240); UcCBM5 (UHOR_00973) interacted with serine/threonine protein phosphatase (UHOR_07708), ALG1-beta-mannosyltransferase (UHOR_06695), and glycogen [starch] synthase (UHOR_01531); and UcCBM8 (UHOR_04077) interacted with the Fe-S protein (UHOR_06641), cytochrome b5 family protein (UHOR_02592), and Fe-S cluster assembly protein (DRE2) (Figure 7). In this interaction network, UcCBM1 (UHOR_06976) and UcCBM5 (UHOR_00973) form the core of the interactions (Figure 7). These functional partner proteins may work together with UcCBMs to regulate the growth and development of pathogens, and are involved in the interactions between pathogens and their hosts.

## 3. Discussion

Foxtail millet kernel smut (FMKS) has become an important factor in restricting the production of foxtail millet in the world [20]. Recently, although the genome sequencing of *U. crameri* has been completed [16], the study of pathogenicity function genes has been limited. Many studies have confirmed that CBM proteins of plant pathogens can act as effectors and play crucial roles in the interactions between pathogens and their hosts [21,22]. In this study, 11 CBM genes were identified via a bioinformatics analysis based on the genome data of *U. crameri*. The number of UcCBM genes was less than those of the necrotrophic fungus *Rhizoctonia solani* [23] and the hemibiotroph *Magnaporthe grisea* [24], which may be due to the fact that the biotrophic *U. crameri* did not destroy the host cell wall during the infection.

Only five UcCBM proteins have the conserved domain of typical CBM or GH family proteins; however, the CDS of UcCBM4 and UcCBM6 contains a carbohydrate-binding site. Furthermore, several LysM domains have a chitin-binding ability, and are known as CBM protein family 50 domains [25]. The proteins within the LysM domain directly play a role either in the immunity regulation of pattern recognition receptors (PRRs) or as effectors in pathogenic fungi that suppress plant immunity [26,27,28]. Our result indicates that UcCBM10 contains two LysMs conserved domains; we speculate that this gene may act as an effector and play crucial roles in the *U. crameri*–host interaction. VdEG3, an effector of *V. dahlia*, which has both the CBM1 and GH12 conserved domains, could trigger cell death in *N. benthamiana* via its GH12 (VdEG3^GH12^) domain [8]. Interestingly, the CMB1 domain of VdEG3 could suppress the cell death-inducing activity of VdEG3^GH12^. We also found that UcCBM10 containing both CBM and GH conserved domains and their domain features was consistent with VdEG3. Thus, UcCBM10 and VdEG3 may have similarities in function when it comes to pathogen–host interactions.

Promoter cis-acting element analysis is key to studying gene function [29]. Previous studies showed that CBM proteins could provide energy for plant growth and development and stress response by becoming involved in starch biosynthesis and degradation [30,31,32]. For instance, the rice CBM protein FLO6 can bind to starch by its CBM48 domain and plays a critical role in starch synthesis [33]. Our results showed that the promoter region of the UcCBM gene family possessed more hormone response and stress response elements, indicating that the UcCBMs were involved in fungi growth and development and the hormonal regulation of fungi biological processes. Interestingly, we also found that UcCBM1 and UcCBM5 interacted with glycogen [starch] synthase (UHOR_01531), which involved starch synthesis through PPI analysis. However, the functions of these UcCBM genes and how UcCBM regulates the growth and development of *U. crameri* need to be further studied.

The CBM protein family plays an essential role in hydrolyzing the wall polysaccharides of a plant [34]. The GO enrichment analysis results of this study showed that the function of UcCBM genes is mainly related to carbohydrate metabolic processes, and in particular the glycogen metabolic process, arabinose metabolic process, L-arabinose metabolic process, and pentose metabolic process. We found that most of the UcCBM proteins were downregulated during the early stages of *U. crameri* infection; this may have been due to the fact that *U. crameri*, a biotrophic fungal pathogen, did not break the cell walls of the plant during infection. Furthermore, the expression of effector genes in phytopathogens is typically induced during their infection of a host plant [35,36,37,38]. In our study, UcCBM8, UcCBM4, and UcCBM1 were upregulated during *U. crameri* infection, similarly to most virulence factors of phytopathogenic fungi, such as SCRE1 and UvGHF1 of *Ustilaginoidea virens*, which causes rice false smut [39,40]. Thus, we speculate that these proteins may act as an effector and play an important role in *U. crameri* infection.

These findings establish a foundation for developing a deeper understanding of the potential roles of CBM genes in fungi. Whole-genome analysis enables us to preliminarily characterize the UcCBMs in *U. crameri*. Our results are mainly based on bioinformatics analysis, and the true role of UcCBMs in *U. crameri* growth and pathogenicity still needs more experiments to be verified. The molecular mechanism of UcCBM genes involved in interactions between *U. crameri* and its host also need further exploration. For example, determining the heterologous expression of these UcCBM proteins in plants is a good method for validating their virulence function [41,42]. Furthermore, developing the small interfering RNAs (siRNAs) of these UcCBM genes will also help us in understanding the influence of UcCBMs on plant immunity and may lay a foundation for the potential practical application of UcCBMs in foxtail millet anti-disease methods and breeding [43,44,45].

## 4. Materials and Methods

### 4.1. Identification and Physicochemical Property Analysis of UcCBM Family Genes

The *U. crameri* SCZ-6 genomic data and relevant annotation files were obtained from our previously reported study [16]. We used the CAZy database (http://www.cazy.org/, accessed on 5 September 2024) with an e-value of less than 1 × e^−5^ to search the CMB proteins in the *U. crameri* genome. The online ExPASy software (https://web.expasy.org/protparam/, accessed on 5 September 2024) was used to predict the physicochemical properties of the UcCBM proteins [46]. The website https://wolfpsort.hgc.jp/, accessed on 5 September 2024, was used to predict the subcellular localization of the UcCBM proteins.

### 4.2. Multiple Sequence Alignment and Phylogenetic Analysis

The homologous proteins of UcCBMs in *Sporisorium scitamineum*, *U. maydis*, and *U. hordei* were identified by using BLAST with the NCBI website (https://www.ncbi.nlm.nih.gov/, accessed on 5 September 2024). All 11 UcCBM family proteins and their homologous proteins were aligned using Clustal W and default parameters. The phylogenetic tree was constructed using the MEGA 7.0 software with a Neighbor-Joining (NJ) method.

### 4.3. Gene Structure, Protein Motif, and 3D Structure Analysis

GSDS (https://gsds.gao-lab.org/index.php, accessed on 5 September 2024) was used to predict the exons or introns of the *UcCBM* genes [47]. The MEME website (http://meme-suite.org, accessed on 5 September 2024) was used to analyze the conservative motifs of the UcCBM proteins, with a pattern count of 10 and other parameters set to default values [48]. The 3D structures of the UcCBMs were created using the website https://www.swissmodel.expasy.org/, accessed on 5 September 2024, [49,50].

### 4.4. Promoter Cis-Acting Regulatory Elements Analysis

The promoter sequences 2000 bp upstream of the transcription start site of the *UcCBM* genes were generated from the *U. crameri* SCZ-6 genome sequences [16]. Cis-acting regulatory elements of 11 UcCBM genes were identified through the PlantCARE online network server (http://bioinformatics.psb.ugent.be/webtools/plantcare/html/, accessed on 5 September 2024).

### 4.5. GO Enrichment and Protein–Protein Interaction Analysis

We used the website (https://www.omicshare.com/tools, accessed on 10 September 2024) to perform the GO function enrichment of the UcCBM proteins; and the website https://cn.string-db.org/, accessed on 10 September 2024, was used to identify the partner proteins interacting with the UcCBM family proteins. The smut fungi *U. hordei* was selected as a reference genome.

### 4.6. Analysis of Expression Patterns of UcCBM Family Genes

According to the previous study [16], the transcriptome expression levels of 11 UcCBM genes at different time points (0 h, 12 h, 24 h, and 72 h) after *U. crameri* inoculation were obtained. The expression heat map of 11 UcCBM genes was drawn using the following website (https://www.omicshare.com/tools, accessed on 10 September 2024).

## 5. Conclusions

In summary, the members of the UcCBM proteins were identified and analyzed at the genome-wide level to explore their biological function in interactions between *U. crameri* and plants. A total of 11 UcCBM proteins were identified from the *U. crameri* genome, and their physicochemical properties, evolutionary relationships, and potential biological functions were further predicted and clarified. This study provides a reference for exploring the gene functions of UcCBMs, and lays a foundation for future efforts to detect the molecular mechanisms of interactions between *U. crameri* and foxtail millet.

## Figures and Tables

**Table 1:** Nomenclature and characteristics of the predicted carbohydrate binding modules (CBMs) in *U. crameri*.

Proposed Gene Name	Gene ID	Superfamily	CDS Length (bp)	Protein Length (aa)	Mw (KDa)	pI	GRAVH	Predicted Subcellular Localization
*UcCBM1*	Ustilago0G003590	CBM48	2094	698	79.29	5.94	−0.35	mitochondrion
*UcCBM2*	Ustilago0G007370	CBM4	2097	699	76.23	5.82	−0.24	extracellular
*UcCBM3*	Ustilago0G009390	CBM43	1731	577	60.42	4.6	0.008	extracellular
*UcCBM4*	Ustilago0G030860	CBM18	1137	379	41.65	8.83	−0.288	extracellular
*UcCBM5*	Ustilago0G032100	CBM21	3021	1007	107.5	8.09	−0.602	nucleus
*UcCBM6*	Ustilago0G039330	CBM18	2148	716	76.63	8.71	−0.512	extracellular
*UcCBM7*	Ustilago0G044030	CBM63	1662	554	57.78	5.48	−0.766	extracellular
*UcCBM8*	Ustilago0G046260	CBM35	1410	470	50.7	4.57	−0.186	extracellular
*UcCBM9*	Ustilago0G049680	CBM35	1827	609	64.41	5.09	−0.204	extracellular
*UcCBM10*	Ustilago0G052360	CBM50	771	257	27.65	5.95	−0.333	extracellular
*UcCBM11*	Ustilago0G057940	CBM32	513	171	18.42	4.92	−0.233	extracellular

ID: identity; bp: base pair; aa: amino acid; KDa: kilo Dalton; pI: isoelectric point; Mw: molecular weight; GRAVH: grand average of hydropathicity.

## Data Availability

The original contributions presented in the study are included in the article; further inquiries can be directed to the corresponding authors.

## Supplementary Materials

The following supporting information can be downloaded at: https://www.mdpi.com/article/10.3390/ijms252111790/s1.
